# Comparative Effects of Fruit-Derived Pectins on the Stability and Antioxidant Activity of Bilberry Anthocyanin–Pectin Complexes

**DOI:** 10.3390/foods15183312

**Published:** 2026-09-18

**Authors:** Zhilin Gan, Kaiyi Xu, Baoqing Zhu, Yu Shuai, Honglin An, Sijia Wu, Yixuan Li, Qianyun Han

**Affiliations:** 1 College of Biological Sciences and Technology, Beijing Forestry University, Beijing 100083, China; 2 Beijing Key Laboratory of Forest Food Processing and Safety, Beijing Forestry University, Beijing 100083, China; 3 Hebei Provincial Key Laboratory of Sustainable Utilization and Development of Forest Food Resources, Beijing Forestry University, Beijing 100083, China

**Keywords:** bilberry anthocyanins, pectin, pectin–anthocyanin complex, anthocyanin stability, antioxidant activity

## Abstract

European bilberry anthocyanins are promising natural pigments and bioactive compounds, but their application in food systems is limited by their sensitivity to environmental stresses. This study compared apple pectin (AP), citrus pectin (CP), and blueberry pectin (BP) for their effects on the association, stability, and antioxidant activity of bilberry anthocyanins. The optimized complexation conditions were an anthocyanin-to-pectin mass ratio of 1:2.5, pH 3.0, and 80 min. The pectins differed in monosaccharide composition, with (Gal + Ara)/Rha ratios of 2.06, 2.68, and 3.88 for AP, CP, and BP, respectively. Under the these conditions, the binding percentage followed the order AP-A > CP-A > BP-A. FT-IR, XRD, and SEM analyses showed changes in functional group regions, diffraction patterns, and surface morphology after complex formation, consistent with non-covalent association. AP-A showed the highest protective effect among the tested complexes, increasing anthocyanin retention from 48.52% to 64.42% after heating at 90 °C for 5 h and from 8.90% to 37.18% after 5 days of indoor natural light exposure. In the antioxidant assays, AP-A reduced the DPPH and ABTS IC_50_ values from 46.64 to 31.12 μg/mL and from 0.90 to 0.372 mg/mL, respectively. These findings provide useful information for understanding the relationship between pectin compositional features and anthocyanin stabilization, and may support the rational selection of pectin matrices for anthocyanin-rich food systems.

## 1. Introduction

European bilberry (*Vaccinium myrtillus* L.) is a wild, low-growing deciduous shrub mainly distributed in northern Europe and other high-latitude regions. Due to its specific ecological requirements and limited large-scale cultivation, bilberry remains an economically valuable wild berry species [1,2]. Bilberries are particularly valued for their high content and diverse profile of anthocyanins, which have been reported to account for a large proportion of the total phenolic extract and to be present at higher levels than in many cultivated blueberries [3,4]. This complex anthocyanin profile, comprising several derivatives including delphinidin, cyanidin, petunidin, peonidin, and malvidin glycosides, has been associated with a range of biological activities, including antioxidant, anti-inflammatory, and metabolic regulatory effects [5,6,7,8,9,10,11].

Despite their potential as natural colorants and functional ingredients, the application of bilberry anthocyanins in food systems is limited by their chemical instability. Anthocyanins are flavonoid pigments characterized by the 2-phenylbenzopyrylium skeleton, and their color and stability are strongly affected by environmental factors such as pH, temperature, light, oxygen, metal ions, and coexisting food components [12,13,14]. Under unfavorable conditions, anthocyanins may undergo structural transformations, including hydration of the flavylium cation, formation of colorless carbinol pseudobases or chalcones, and subsequent degradation reactions. These changes can lead to color fading and reduced functional performance of anthocyanin-rich extracts. Therefore, improving anthocyanin stability under food processing and storage conditions has become an important topic in food chemistry and functional food research [15,16].

Several strategies have been explored to improve anthocyanin stability, including co-pigmentation, encapsulation, emulsion-based delivery systems, and complexation with proteins or polysaccharides [17,18]. These approaches can improve pigment retention by modifying the local chemical environment, reducing exposure to external stressors, or promoting stabilizing intermolecular interactions. Recent studies have further shown that complexation with polysaccharides, such as pectin, inulin, starch, and cellulose, can improve anthocyanin stability, with pectin showing particularly favorable complexation and protective effects in some systems [19]. However, some encapsulation and composite delivery systems require multi-component formulations, specific processing conditions, or additional wall materials, which may limit their practical use in simple food matrices. Moreover, many studies focus on the overall protective effect of the formulation, whereas the relationship between specific polysaccharide structural features and direct anthocyanin association is still not fully understood. Thus, there is still a need to develop simple food-grade systems and to clarify how structural differences in stabilizing polymers influence anthocyanin binding and stability.

Pectin is a widely used food-grade polysaccharide and has attracted attention as a stabilizing material for anthocyanins. It is a complex acidic heteropolysaccharide mainly composed of homogalacturonan (HG) regions and rhamnogalacturonan-I (RG-I) regions, with neutral sugar side chains such as arabinose (Ara) and galactose (Gal) associated with RG-I domains [20]. The functional properties of pectin are related to its botanical source and structural characteristics, including the degree of esterification, molecular weight, galacturonic acid (GalA)-rich regions, and neutral sugar side-chain composition. Previous studies have shown that pectin can interact with anthocyanins through non-covalent interactions, including hydrogen bonding, electrostatic interactions, and hydrophobic interactions, thereby improving anthocyanin stability under certain conditions [21,22]. Moreover, pectin structural properties, including the degree and pattern of esterification, have been shown to affect anthocyanin binding, while blueberry pectin has also been reported to interact with cyanidin-3-O-glucoside [22,23]. Nevertheless, although pectin–anthocyanin interactions have been investigated, the role of compositional differences among commercial fruit-derived pectin samples in bilberry anthocyanin association and stabilization remains to be further clarified.

In this study, apple pectin (AP), citrus pectin (CP), and blueberry pectin (BP) were selected as model pectins for comparative investigation. AP and CP represent common fruit-derived commercial pectins used in food systems, whereas BP was included as a berry-derived pectin with closer botanical relevance to bilberry and previously reported capacity to interact with anthocyanins [20,24]. This comparison also allowed us to examine whether compositional differences among the three commercial pectin samples, particularly monosaccharide-derived features related to rhamnose (Rha)-containing regions and neutral sugar side chain-related components, are associated with bilberry anthocyanin association and stabilization. Based on this rationale, the complexation conditions of bilberry anthocyanins with AP, CP, and BP were optimized, and the resulting complexes were characterized using Fourier transform infrared spectroscopy (FT-IR), X-ray diffraction (XRD), and scanning electron microscopy (SEM). Their stability under thermal, light, metal ion, preservative, oxidative, and reductive stresses was then evaluated, together with antioxidant activity. This work provides a comparative basis for selecting suitable pectin sources to improve the stability of bilberry anthocyanins in food systems and offers further insight into the association between pectin compositional characteristics and anthocyanin-binding, stabilization, and antioxidant performance.

## 2. Material and Methods

### 2.1. Material

Bilberry anthocyanin extracts were purchased from Xi’an Xiaocao Plant Technology Co., Ltd. (Xi’an, China). The commercial bilberry extract was prepared by cold maceration and standardized to approximately 25% anthocyanins. Apple pectin and citrus pectin were both purchased from Yantai DSM Andeli Pectin Co., Ltd. (Yantai, China). Blueberry pectin was purchased from Guangzhou Xichu Biotechnology Co., Ltd. (Guangzhou, China). Sodium benzoate and potassium sorbate (food-grade) were purchased from Beijing Solarbio Technology Co., Ltd. (Beijing, China). All other chemicals and solvents were of analytical grade.

### 2.2. High-Performance Liquid Chromatography-Tandem Mass Spectrometry (HPLC-MS/MS) Analysis of Anthocyanin Composition

Anthocyanin composition was analyzed using a high-performance liquid chromatography system (Waters) coupled with a Q Exactive mass spectrometer (Q Exactive, Thermo Scientific, Waltham, MA, USA), following the method of Colak et al. (2017) with some modifications [25]. Chromatographic separation was performed on a Waters HSS T3 column (100 × 1 mm, 1.8 μm) at 40 °C. The mobile phases consisted of 0.1% formic acid in water (A) and 0.1% formic acid in acetonitrile (B). The gradient elution was performed at a flow rate of 0.3 mL/min as follows: 0–2 min, 5% B; 2–15 min, 5–30% B; 15–15.1 min, 30–95% B; 15.1–20 min, 95% B; 20–20.1 min, 95–5% B; 20.1–26 min, 5% B. The injection volume was 2 μL. Mass spectrometry was performed using a Q Exactive mass spectrometer (Thermo Scientific) equipped with an electrospray ionization source operating in positive ion mode. Full-scan MS spectra were acquired over an *m*/*z* range of 200–700, followed by data-dependent MS/MS (dd-MS^2^) acquisition for fragmentation of selected precursor ions by higher-energy collisional dissociation (HCD). Data were processed using Xcalibur software (version 4.5), with a mass tolerance of 5 ppm. Individual anthocyanins were tentatively identified based on their retention times, precursor ions, characteristic fragment ions, typical neutral losses, and comparison with published fragmentation data.

### 2.3. Monosaccharide Composition Analysis of Pectin Samples

The monosaccharide profiles of commercial AP, CP, and BP were determined by ion chromatography (ICS5000, Thermo Scientific) using an electrochemical detector. According to the method of Song et al. [26], samples were hydrolyzed with 2 M trifluoroacetic acid at 120 °C for 2 h, followed by evaporation and reconstitution in ultrapure water. Samples were then separated on a Dionex™ CarboPac™ PA20 column (150 × 3.0 mm, 10 μm) at 30 °C. The injection volume was 5 μL. The mobile phases included ultrapure water (A), 0.1 M NaOH (B), and 0.1 M NaOH containing 0.2 M NaAc (C) at a flow rate of 0.5 mL/min. Monosaccharide contents were expressed as μg/mg sample, and the derived ratios of rhamnose to galacturonic acid (Rha/GalA) and galactose plus arabinose to rhamnose [(Gal + Ara)/Rha] were used as compositional descriptors for comparing the three commercial pectin samples.

### 2.4. Determination of Anthocyanin Content

The total anthocyanin content was quantified using the pH differential method as described by Lee et al. and Harakotr et al. with slight modifications [27,28]. Samples were appropriately diluted with two different buffers: 0.025 M HCl-KCl buffer (pH 1.0) and 0.4 M CH_3_COONa buffer (pH 4.5). After equilibration for 15 min, the absorbance of each solution was measured at 520 nm and 700 nm by using an ultraviolet-visible (UV-Vis) spectrophotometer (UV-4800, Unicosh, Shanghai, China). The anthocyanin concentration was calculated according to the following equation:

$$\mathrm{Anthocyanin}\;\mathrm{content}\;(\mathrm{mg}/L)\;=\;\;\frac{A\;\times \;DF\;\times \;Mr\;\times \;1000}{\varepsilon \;\times \;L}$$

where A = (A_520nm_ − A_700nm_)_pH1.0_ − (A_520nm_ − A_700nm_)_pH4.5_. DF is the dilution factor; Mr represents the molecular weight of cyanidin-3-glucoside (449.2 g/mol); ε is the molar absorptivity of cyanidin-3-glucoside (26,900 L·mol^−1^·cm^−1^); L is the cuvette thickness (optical path, 1 cm). The total anthocyanin content was expressed as cyanidin-3-glucoside equivalents.

### 2.5. Preparation and Optimization of Pectin–Anthocyanin Complexes

The preparation of pectin–anthocyanin complexes was optimized by investigating three key variables: anthocyanin-to-pectin mass ratio (A:P), pH, and complexation time. In all experiments, bilberry anthocyanin extract was dissolved in citrate buffer to a concentration of 4 mg/mL.

#### 2.5.1. Ratio

To determine the effect of the mass ratio, the anthocyanin solution (pH 3.0) was mixed with an equal volume of pectin solutions at concentrations of 2, 4, 6, 8, and 10 mg/mL. This resulted in a series of anthocyanin-to-pectin mass ratios (A:P) of 1:0.5, 1:1, 1:1.5, 1:2, and 1:2.5, respectively. After equal-volume mixing, the final anthocyanin concentration was 2 mg/mL, and the final pectin concentrations were 1, 2, 3, 4, and 5 mg/mL, respectively. Each mixture was homogenized via magnetic stirring at 500 rpm for 80 min.

#### 2.5.2. pH

The influence of pH was evaluated by dissolving bilberry anthocyanin powder at 4 mg/mL in sodium citrate buffer solutions with pH values adjusted to 1.0, 2.0, 3.0, 4.0, and 5.0. These solutions were then mixed with an equal volume of pectin solution at 10 mg/mL, corresponding to a fixed anthocyanin-to-pectin mass ratio (A:P) of 1:2.5. After equal-volume mixing, the final anthocyanin and pectin concentrations were 2 and 5 mg/mL, respectively. The systems were stirred at 500 rpm for 80 min.

#### 2.5.3. Complexation Time

Based on the optimal mass ratio (1:2.5) and pH (3.0), the effect of complexation time was investigated. The mixtures were subjected to magnetic stirring at 500 rpm for varying durations of 40, 60, 80, 100 and 120 min. Following optimization, the complexes prepared under the selected conditions were collected for further characterization.

#### 2.5.4. Calculation of Binding Percentage

The binding percentage was calculated based on the reduction in free anthocyanin concentration after complexation. This value, therefore, represents an indirect estimate of anthocyanin association with pectin based on the decrease in free anthocyanins remaining in the supernatant, rather than a direct measurement of binding affinity. Briefly, the mixed solution was centrifuged at 10,000× *g* for 20 min at 4 °C, and the anthocyanin concentration in the supernatant was determined using the pH differential method. The binding percentage was calculated as follows: binding percentage (%) = [(C_0_ − C_free_)/C_0_] × 100, where C_0_ is the initial anthocyanin concentration in the mixed system before complexation, and C_free_ is the concentration of unbound anthocyanins in the supernatant after complexation.

### 2.6. Structural and Morphological Characterization

#### 2.6.1. FT-IR Spectroscopy

The intermolecular interactions between bilberry anthocyanins and pectins were analyzed using an FT-IR spectrometer (Nicolet iS5, Thermo Fisher Scientific). Freeze-dried samples (2 mg) were blended with spectroscopic-grade potassium bromide (KBr) (198 mg), finely ground, and compressed into translucent pellets. Spectra were acquired over a wavenumber range of 4000–400 cm^−1^ at a resolution of 4 cm^−1^ with an average of 32 scans per spectrum. For comparative interpretation, the spectra were baseline-corrected and normalized before the major functional group regions were compared.

#### 2.6.2. XRD

The crystalline properties of the lyophilized powders were evaluated using an X-ray diffractometer (XRD-7000S, Shimadzu Corporation, Kyoto, Japan). The analysis utilized a Cu-Kα radiation source (λ = 0.15418 nm) operating at a tube voltage of 40 kV and a current of 40 mA. Diffraction patterns were recorded across a 2θ range of 5–90° with a continuous scanning rate of 5°/min.

#### 2.6.3. SEM

The surface morphology of the pectin–anthocyanin complexes (AP-A, CP-A, and BP-A) was visualized via SEM (Regulus8100, Hitachi Limited, Tokyo, Japan). Prepared samples were fixed onto aluminum stubs using double-sided conductive adhesive tape and sputter-coated with a thin layer of platinum to enhance conductivity. Observations were conducted at an accelerating voltage of 3.0 kV, and representative micrographs were captured at magnifications of 100× and 300×, respectively.

### 2.7. Stability Assessment Under Environmental Stresses

For all stability assays, anthocyanin retention was calculated based on the pH differential absorbance obtained according to Section 2.4. Retention was calculated as retention (%) = (A_t_/A_0_) × 100, where A_0_ is the initial pH differential absorbance before treatment, and A_t_ is the pH differential absorbance after treatment at time t.

#### 2.7.1. Thermal Stability

Samples of free bilberry anthocyanins and pectin–anthocyanin complexes were transferred into sealed 10 mL centrifuge tubes and incubated in a water bath at 50, 70, and 90 °C for 5 h. Aliquots were collected at 0, 1, 2, 3, 4, and 5 h, immediately cooled to room temperature, and subjected to subsequent analysis.

#### 2.7.2. Photostability

Samples were stored under three illumination conditions for 5 days: indoor natural light on a laboratory windowsill with an average daytime ambient temperature of approximately 25 °C, incandescent light in an incubator, and darkness by wrapping them with aluminum foil. Samples were collected every 24 h. The degradation kinetics were analyzed using a first-order kinetic model:

ln(*A_t_*/*A*_0_) = −*kt*

t_1/2_ = ln2/*k*

where A_t_ is the pH differential absorbance after treatment at time t, A_0_ is the initial pH differential absorbance, *k* is the first-order degradation rate constant, and t_1/2_ is the half-life.

#### 2.7.3. Chemical Stability

The stability of free anthocyanins and pectin–anthocyanin complexes was evaluated against various chemical agents. (1) Metal ions: Samples were mixed with equal volumes of 0.1 mol/L solutions of NaCl, CaCl_2_, CuSO_4_, FeCl_3_, and Zn(CH_3_COO)_2_ and incubated in the dark for 2 h. (2) Preservatives: Samples were mixed with equal volumes of sodium benzoate or potassium sorbate solutions (final concentrations: 0.05%, 0.1%, 0.2%, and 0.5%) and stored in the dark for 2 h. (3) Redox agents: H_2_O_2_ and Na_2_SO_3_ were added to the samples at a final concentration of 0.5% (*v*/*v*) and 0.5% (*w*/*v*), respectively, and samples were collected at 15 min intervals for 1 h for H_2_O_2_ treatment and at 30 min intervals for 2 h for Na_2_SO_3_ treatment.

### 2.8. Evaluation of Antioxidant Activity

The antioxidant activities were evaluated via 2,2-diphenyl-1-picrylhydrazyl (DPPH) radical scavenging, 2,2′-azino-bis(3-ethylbenzothiazoline-6-sulfonic acid) radical cation (ABTS•^+^) scavenging, and ferric reducing antioxidant power (FRAP) assays, with ascorbic acid (vitamin C, VC) as the positive control. Free bilberry anthocyanins, pectin–anthocyanin complexes, and VC were prepared at the same mass concentrations specified for each assay. All spectrophotometric measurements were conducted using a UV-Vis spectrophotometer.

DPPH assay: The DPPH radical scavenging activity was determined according to the methods of Li et al. and Pantelidis et al. with minor modifications [11,29]. Sample solutions were prepared at different mass concentrations (20, 40, 60, 80, and 100 μg/mL), mixed with 0.1 mM DPPH solution at a volume ratio of 1:1, and reacted for 30 min in the dark. The absorbance was recorded at 517 nm. The DPPH radical scavenging activity was calculated as follows:

DPPH radical scavenging activity (%) = [(A_0_ − (A_1_ − A_2_))/A_0_] × 100

where A_0_ is the absorbance of the blank group containing DPPH solution without sample, A_1_ is the absorbance of the sample solution mixed with DPPH solution, and A_2_ is the absorbance of the sample solution without DPPH solution. The antioxidant potency was further quantified by the IC_50_ value, defined as the sample concentration required to scavenge 50% of the DPPH radicals, which was derived from the non-linear regression of the scavenging activity–concentration curve.

ABTS•^+^ assay: The ABTS•^+^ radical scavenging activity was determined according to the methods of Re et al. and Li et al. with slight modifications [29,30]. The ABTS•^+^ working solution was prepared by dissolving 0.0192 g ABTS and 0.0033 g potassium persulfate (K_2_S_2_O_8_) in 5 mL deionized water, followed by incubation in the dark for 12–16 h at room temperature. Before use, the working solution was diluted with absolute ethanol to an absorbance of 0.70 ± 0.02 at 734 nm. Sample solutions were prepared at different mass concentrations (0.1, 0.2, 0.3, 0.4, and 0.5 mg/mL) and mixed with the ABTS•^+^ working solution at a volume ratio of 1:39 and incubated for 10 min at room temperature. The absorbance was determined at 734 nm, and IC_50_ values were calculated based on the dose–response curves. The ABTS•^+^ radical scavenging activity was calculated using the following equations:

ABTS•^+^ radicl scavenging activity (%) = [(A_blank_ − A_sample_)/A_blank_] × 100

where A_blank_ is the absorbance of the blank control containing the ABTS•^+^ working solution without a sample, and A_sample_ is the absorbance of the sample solution mixed with the ABTS•^+^ working solution. The scavenging efficiency was expressed as the IC_50_ value, defined as the sample concentration required to scavenge 50% of the ABTS•^+^ radicals, and was calculated from the dose–response curve of scavenging activity versus sample concentration.

FRAP assay: The FRAP assay was measured according to the method of Benzie & Strain (1999) with slight modifications [31]. The FRAP reagent was freshly prepared by mixing acetate buffer (300 mmol/L, pH 3.6), FeCl_3_ solution (20 mmol/L), and TPTZ solution (10 mmol/L, prepared in 40 mmol/L HCl) at a volume ratio of 10:1:1. Sample solutions were prepared at different mass concentrations (20, 40, 60, 80, and 100 μg/mL) and mixed with freshly prepared FRAP reagent by adding 0.02 mL of sample solution to 3 mL FRAP reagent, and incubated at 37 °C for 10 min. The absorbance was recorded at 593 nm. The FRAP value was calculated using an FeSO_4_·7H_2_O standard curve over the concentration range of 0.15 to 1.5 mmol/L (y = 0.2361x + 0.0323, R^2^ = 0.996), and the results were expressed as μmol Fe^2+^ equivalents/L.

### 2.9. Statistical Analysis

All experiments were performed in triplicate, and the results were expressed as mean values ± standard deviation (SD). Statistical significance (*p* < 0.05) was determined using one-way ANOVA followed by Duncan’s multiple range test for single-factor comparisons and two-way ANOVA for evaluating the main and interaction effects of sample type and treatment concentration or time. Statistical analyses were performed using SPSS 26.0. Data visualization was performed using Origin 9.1.

## 3. Results and Discussion

### 3.1. Structural Characterization of Bilberry Anthocyanins and Pectins

#### 3.1.1. Spectroscopic Characterization and Anthocyanin Composition Analysis of Bilberry Extract

The optical properties of bilberry anthocyanins were initially evaluated via full-wavelength UV-Vis scanning (200–800 nm). As illustrated in Figure S1, two typical absorption maxima were observed: one in the ultraviolet region (310–320 nm) and another in the visible region (500–540 nm). The prominent peak at 518 nm is attributed to the π→π* transition associated with the flavylium chromophore, which is consistent with previous findings for Vaccinium species [32]. This spectral profile confirmed the typical absorption characteristics of bilberry anthocyanins and supported their subsequent quantification using the pH differential method.

To further characterize the anthocyanin composition, HPLC-MS/MS was employed (Table S1). Three representative anthocyanin signals were tentatively assigned based on their mass-to-charge ratios (*m*/*z*), characteristic fragmentation patterns, and comparison with published data. The molecular ion at *m*/*z* 449 [M]^+^ generated a fragment at *m*/*z* 287 through the neutral loss of a hexosyl moiety ([M-162]^+^), and was tentatively assigned to cyanidin-3-O-glucoside. Another peak showed a molecular ion at *m*/*z* 595 [M]^+^ and a secondary fragment at *m*/*z* 287, consistent with the sequential loss of one deoxyhexosyl moiety ([M-146]^+^) followed by one hexose moiety ([M-146-162]^+^), and it was therefore tentatively assigned to cyanidin-3-O-rutinoside. A third component with *m*/*z* 463 [M]^+^ generated a fragment at *m*/*z* 301 through the loss of one hexose moiety ([M-162]^+^), and was tentatively assigned to peonidin-3-O-glucoside. The occurrence of cyanidin-3-O-glucoside and peonidin-3-O-glucoside is consistent with previously reported anthocyanin profiles of *V. myrtillus*, in which cyanidin, delphinidin, petunidin, peonidin, and malvidin glycosides are characteristic constituents [33,34].

From a structural perspective, the detected anthocyanin constituents may provide relevant structural information for interpreting their potential interactions with pectin, because the hydroxylation and methoxylation patterns of the anthocyanin B-ring may influence their interaction with pectin [32]. Specifically, the presence of cyanidin derivatives, which possess two hydroxyl groups on the B-ring, could provide sites for hydrogen bonding with the carboxyl and hydroxyl groups of the pectin backbone [22,35]. Furthermore, sugar moieties, such as glucose and rutinoside, may exert steric effects during the complexation process [36]. The tentative anthocyanin profile provides compositional information that supports the subsequent discussion of pectin–anthocyanin complex formation and stability behavior [22,32,37].

#### 3.1.2. Monosaccharide Composition of Pectin Samples

The monosaccharide composition provides preliminary compositional information on the tested pectin samples, particularly the relative abundance of GalA-rich components and neutral sugar components, which may influence their interaction behavior with bioactive compounds. In this study, the Rha/GalA ratio was used as an approximate compositional indicator to compare the relative abundance of Rha-containing and GalA-containing components among the three commercial pectin samples. As calculated from Table 1, the Rha/GalA ratios for AP, CP, and BP were 0.45, 0.11, and 0.37, respectively, indicating composition-related differences among the three samples. The monosaccharide-derived indicator of neutral sugar side chains, expressed as the (Gal + Ara)/Rha ratio, was 2.06 for AP, 2.68 for CP, and 3.88 for BP (Table 1). Based on these values, AP showed the lowest neutral sugar side-chain indicator, CP was intermediate, and BP showed the highest value among the three samples. Considering the relatively low GalA and comparatively high glucose content observed in some samples, the monosaccharide data were interpreted mainly as comparative compositional descriptors under the present analytical conditions.

From a physicochemical perspective, monosaccharide-derived compositional features can provide information on structural differences among pectin samples that may influence the accessibility of interaction sites [38]. The relatively lower (Gal + Ara)/Rha value of AP suggests a lower neutral sugar side-chain indicator among the three samples, which may favor greater accessibility of pectin functional groups involved in anthocyanin association [22,38]. In contrast, the higher (Gal + Ara)/Rha values observed in CP and BP may indicate higher neutral sugar side-chain indicators, which could be associated with greater steric effects during pectin–anthocyanin association. Previous studies have also shown that pectin structural characteristics, including esterification pattern and degree of methyl esterification, can markedly influence anthocyanin binding affinity [22]. Therefore, variations in monosaccharide composition and derived neutral sugar side-chain may represent one of the compositional factors associated with the different anthocyanin-binding behaviors observed among the pectin samples.

### 3.2. Effects of Binding Conditions on Pectin–Anthocyanin Complexes

**Figure 1:**
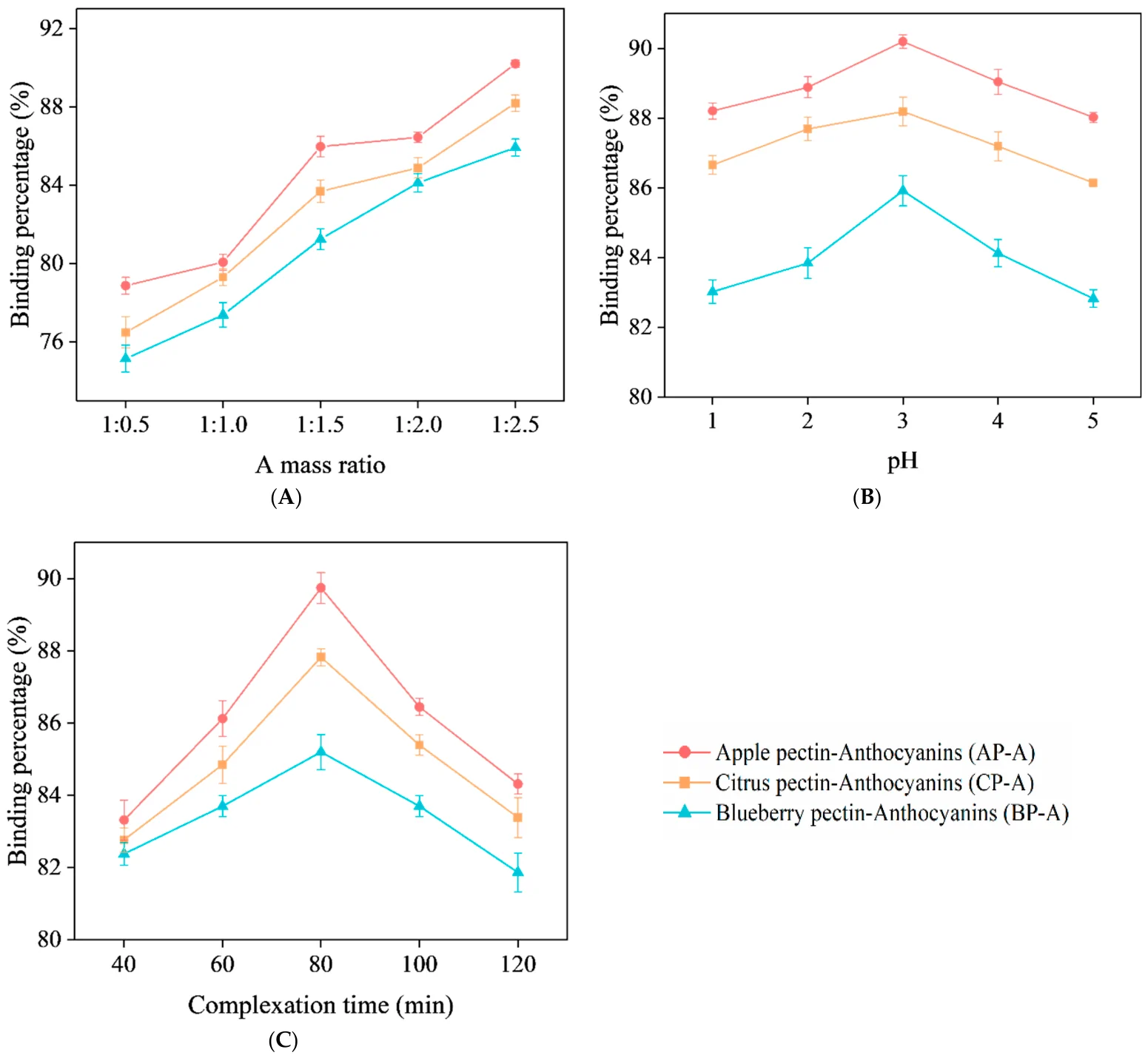
Effects of complexation conditions on the binding percentage of bilberry anthocyanins with different commercial pectin samples: (**A**) Anthocyanin-to-pectin mass ratio; (**B**) pH; (**C**) complexation time.

The binding percentage of bilberry anthocyanins with pectin was influenced by the anthocyanin-to-pectin mass ratio, pH, and complexation time (Figure 1). At a constant initial anthocyanin concentration (4 mg/mL), the binding percentage increased as the A:P ratio changed from 1:0.5 to 1:2.5. This trend is consistent with the findings of Lin et al. [21], suggesting that a higher availability of pectic polysaccharides may provide more accessible interaction sites or functional groups, such as -COOH and -OH, thereby facilitating anthocyanin association within the pectin-containing system. The influence of pH showed a moderate but clear dependence, with the highest binding percentage observed at pH 3.0 for all three pectin types, which is consistent with the observations by Koh et al. [38]. This pH-dependent behavior may be related to the molecular states of both anthocyanins and pectin. At pH 3.0, anthocyanins are relatively enriched in the stable, positively charged flavylium cation form, which may favor electrostatic attraction with partially ionized pectic carboxyl groups [39]. At higher pH values, the gradual transformation of anthocyanins into carbinol pseudobases or chalcones may reduce their association with the pectin matrix. Regarding the complexation time (Figure 1C), the binding percentage increased up to 80 min, followed by a slight decrease at longer times. The decrease observed after 80 min may reflect dynamic rearrangement of the mixed system or partial release of weakly associated anthocyanins during prolonged stirring, rather than continued complex formation. Therefore, 80 min was selected as the optimal complexation time for subsequent experiments.

Notably, the binding percentages consistently followed the order: AP-A > CP-A > BP-A. This difference may be associated with monosaccharide-derived compositional features of the pectin samples. As discussed in Section 3.1.2, AP showed the lowest (Gal + Ara)/Rha ratio, whereas CP and BP showed higher values. Within the three pectin samples examined, the lower (Gal + Ara)/Rha ratio of AP was accompanied by a higher binding percentage, suggesting a possible association between this compositional descriptor and complexation behavior. Previous studies have suggested that neutral sugar side-chain composition may affect steric accessibility of pectin functional groups [40,41]. This observation provides a basis for further investigating the potential role of neutral sugar side-chain characteristics in pectin–anthocyanin complexation. Taken together, these results suggest that the observed complexation behavior may be associated with the compositional characteristics of the tested pectin samples rather than botanical relatedness alone.

### 3.3. Structural Characterization and Surface Morphology of Pectin–Anthocyanin Complexes

#### 3.3.1. FT-IR Analysis

**Figure 2:**
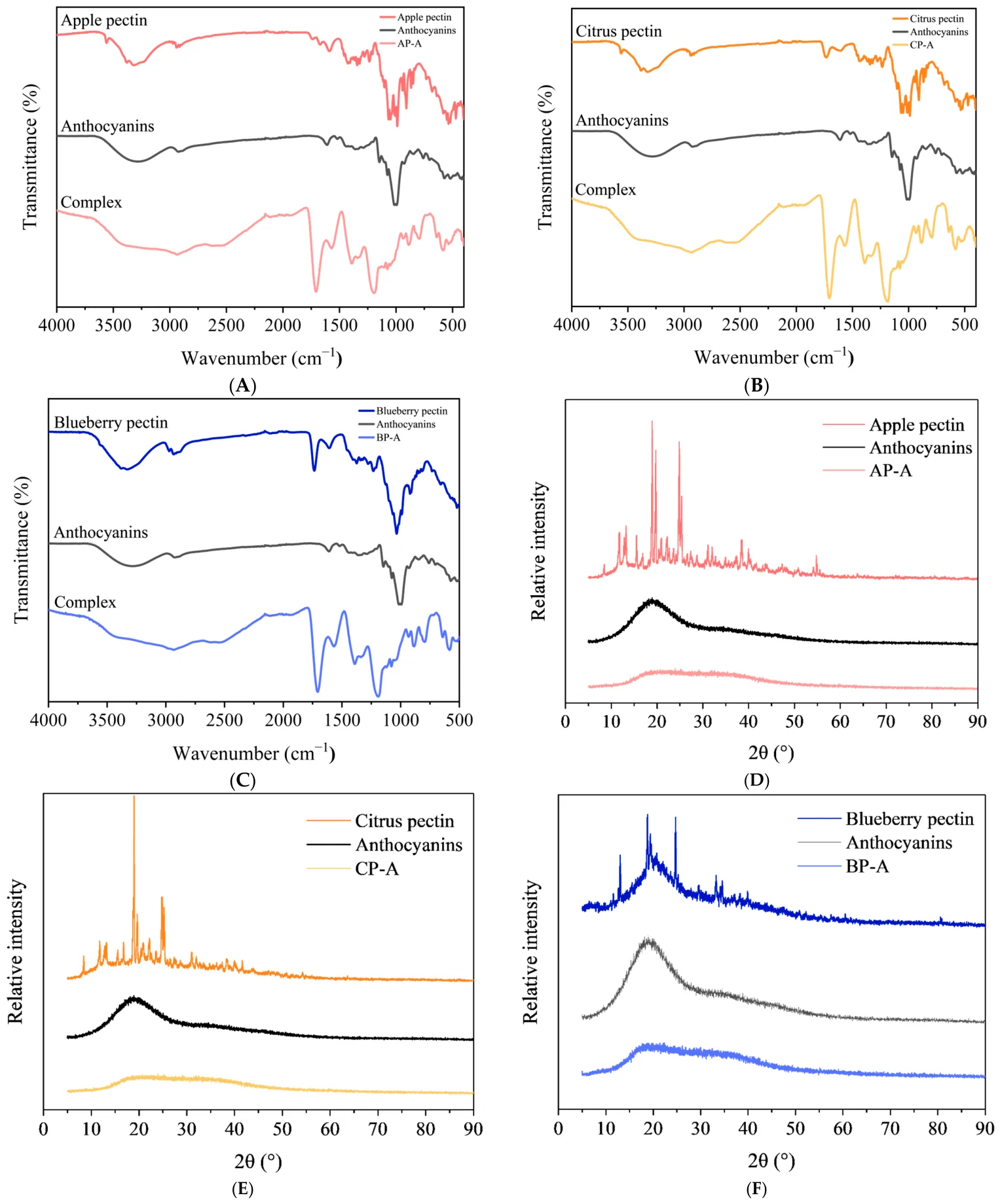
Structural characterization of different pectin–anthocyanin systems. (**A**–**C**) Fourier transform infrared spectra; (**D**–**F**) X-ray diffraction patterns. (**A**,**D**) Apple pectin system; (**B**,**E**) citrus pectin system; and (**C**,**F**) blueberry pectin system.

FT-IR spectroscopy was used to evaluate changes in functional group regions after complex formation (Figure 2A–C). The spectra of all three pectins (AP, CP, and BP) displayed characteristic pectic features: a broad O-H stretching band (3200–3600 cm^−1^), esterified carbonyl absorption (1730–1760 cm^−1^), and carboxylate-related vibrations (1600–1630 cm^−1^). After complexation, changes in peak position and intensity were observed in the O-H stretching region, which may reflect changes in the local environment of hydroxyl groups and are consistent with possible hydrogen bonding interactions. The bands in the carbonyl and carboxylate regions (1740–1600 cm^−1^) also changed after complexation, suggesting that pectic carboxyl groups may participate in the association between anthocyanins and pectins. In addition, no new characteristic absorption bands were observed in the spectra of the complexes, suggesting that the observed complexation was mainly associated with non-covalent interactions rather than the formation of new covalent bonds.

Under the acidic conditions used for complexation (pH 3.0), electrostatic attraction between positively charged flavylium cations and partially dissociated pectic carboxyl groups could be involved in pectin–anthocyanin association, while hydrogen bonding and hydrophobic interactions may also contribute to complex stabilization. This interpretation is consistent with the mechanism proposed by Fernandes et al. [22,39]. The differences in the FT-IR spectra among AP-A, CP-A, and BP-A were generally consistent with the different binding percentages shown in Figure 1, suggesting that the compositional features of the pectin samples may influence their interaction with anthocyanins [33].

#### 3.3.2. XRD Analysis

The physical state of the pectins, anthocyanins, and their complexes was evaluated via XRD (Figure 2D–F). The pectin powders exhibited several diffraction peaks, mainly in the range of 2θ = 20°, indicating the presence of ordered or partially crystalline regions in the pectin powders. In contrast, bilberry anthocyanins showed a broad diffraction band near 2θ = 20°, suggesting a largely amorphous physical state under the tested conditions [42,43,44]. Following complexation, the sharp diffraction peaks of the pectins were weakened, and the complexes showed broader diffraction bands. These changes suggest that the regular arrangement of pectin chains may have been affected after incorporation of anthocyanins, which may be associated with intermolecular interactions between anthocyanins and pectins, including possible hydrogen bonding and electrostatic interactions [42,44]. According to Piacenza et al. [45], polyphenols can be incorporated into polysaccharide matrices and disturb the ordered arrangement of polysaccharide chains. Overall, the weakened diffraction peaks and broader diffraction bands observed in AP-A, CP-A, and BP-A suggest changes in the crystalline characteristics of the pectin matrix after complexation, which is consistent with the FT-IR results.

#### 3.3.3. SEM Analysis

**Figure 3:**
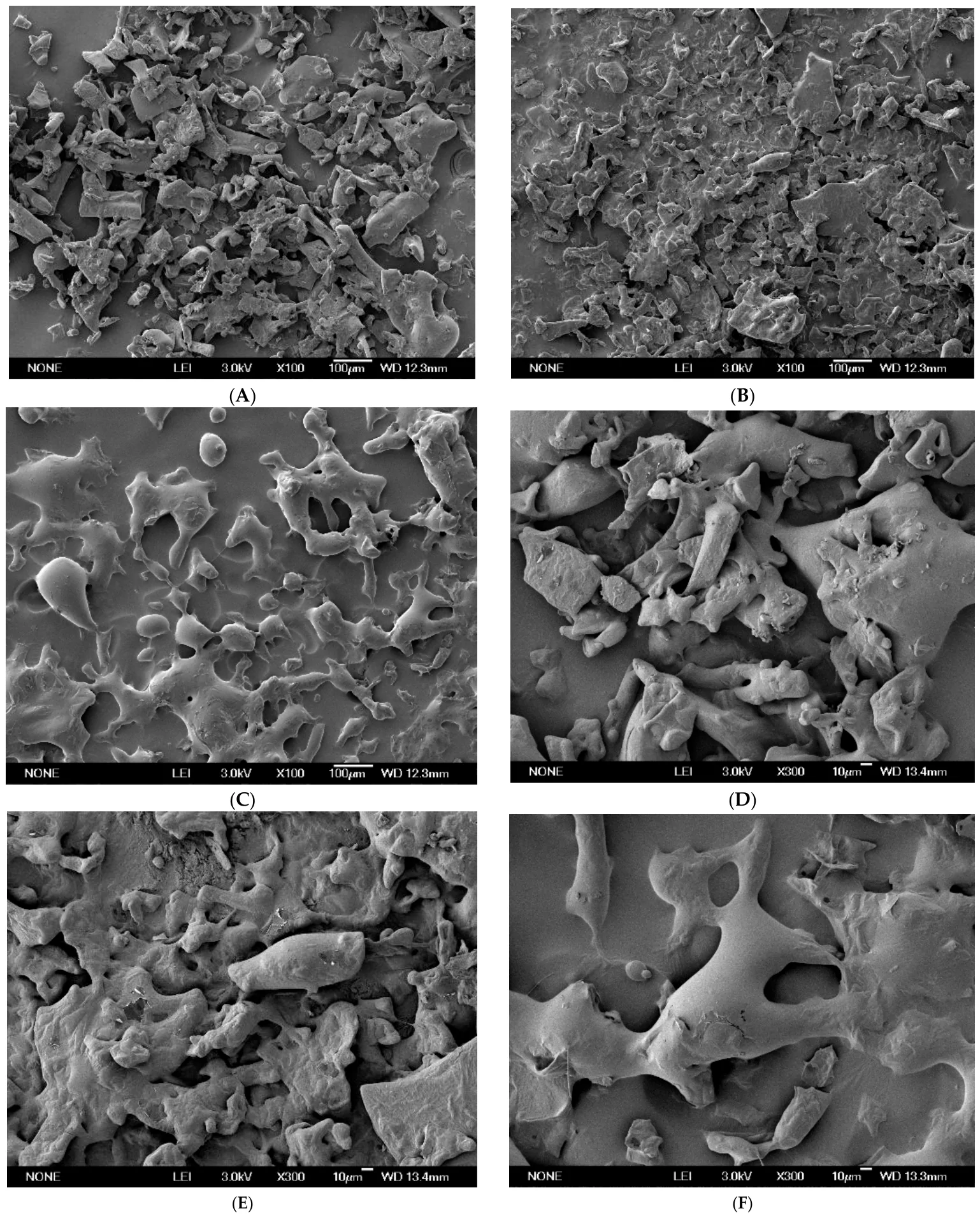
SEM images of the surface morphology of different pectin–anthocyanin complexes: (**A**) AP-A at 100× magnification; (**B**) CP-A at 100× magnification; (**C**) BP-A at 100× magnification; (**D**) AP-A at 300× magnification; (**E**) CP-A at 300× magnification; and (**F**) BP-A at 300× magnification.

The surface morphology of the pectin–anthocyanin complexes was visualized using SEM (Figure 3). All three complexes displayed irregular, aggregated structures with rough surfaces characterized by pores, folds, and fragments. These observations showed morphological differences among AP-A, CP-A, and BP-A, which were consistent with previous reports that polysaccharide-polyphenol complexation can be accompanied by changes in surface morphology [19]. Notably, the morphology varied with the pectin source: AP-A appeared more fragmented and flakier (Figure 3D), CP-A was relatively denser (Figure 3E), while BP-A exhibited a more open, porous network (Figure 3F). These differences may be related to the different compositional features of the pectin samples and their interactions with anthocyanins [35]. The fragmented and flaky morphology of AP-A indicated a different aggregation pattern from CP-A and BP-A, which may be associated with the different binding behavior observed in the AP system. Taken together, the SEM observations revealed discernible differences in the surface morphology of AP-A, CP-A, and BP-A, indicating that the microstructural features of the pectin–anthocyanin complexes varied with the pectin source.

### 3.4. Stability Assessment of Pectin–Anthocyanin Complexes Under Various Environmental Stresses

#### 3.4.1. Thermal Stability

**Figure 4:**
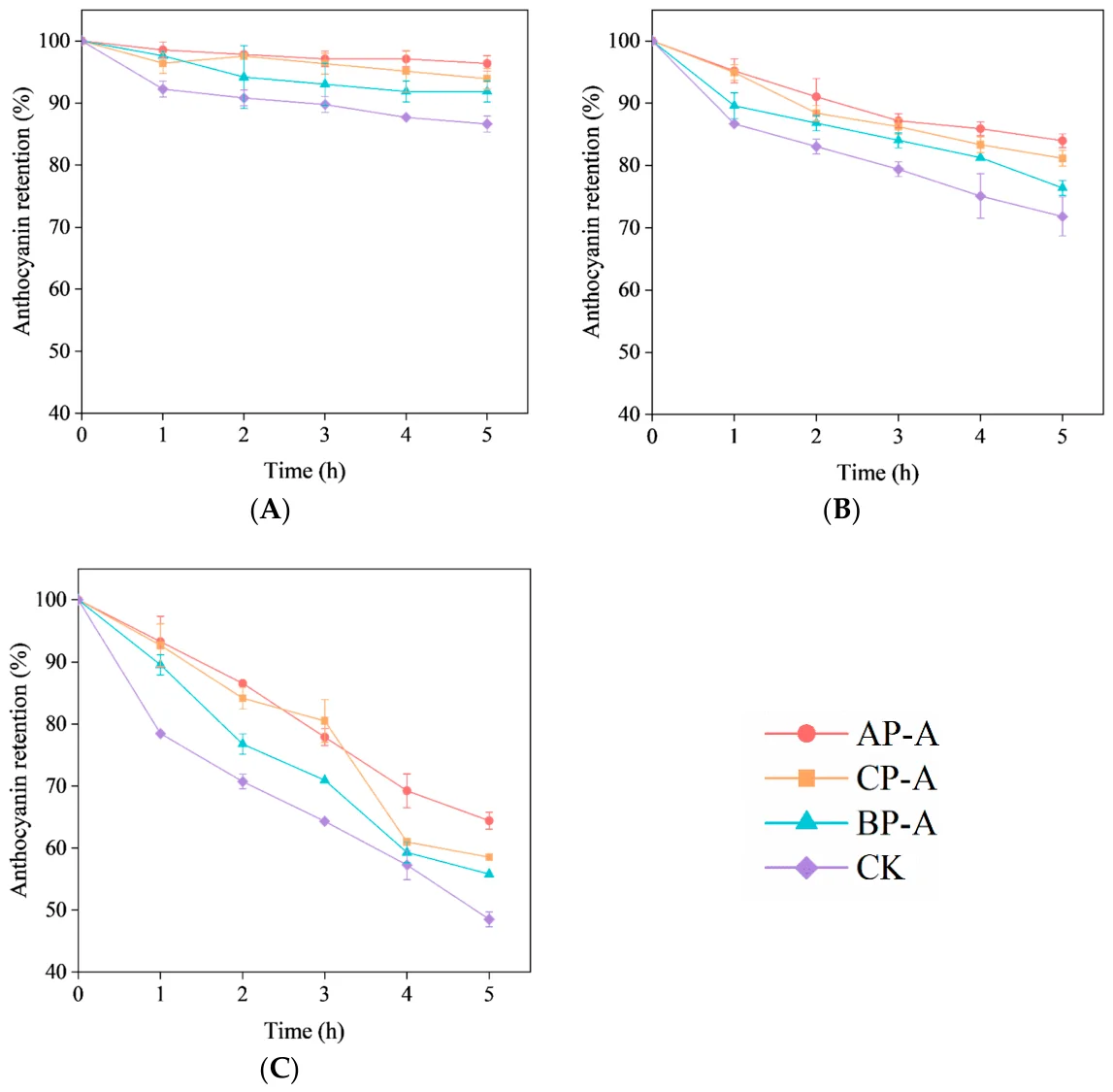
Effect of heating temperature on anthocyanin retention of free bilberry anthocyanins and pectin–anthocyanin complexes. (**A**) 50 °C; (**B**) 70 °C; (**C**) 90 °C.

The thermal stability of bilberry anthocyanins, both in free and complexed forms, was evaluated at 50 °C, 70 °C, and 90 °C for 5 h (Figure 4). At 50 °C (Figure 4A), the three pectin–anthocyanin complexes maintained anthocyanin retention above approximately 91% after 5 h, whereas free anthocyanins exhibited a more pronounced decrease in retention. As the temperature increased to 70 °C (Figure 4B), anthocyanin retention decreased more noticeably than at 50 °C, but the pectin–anthocyanin complexes still retained higher anthocyanin levels than the CK group. At 90 °C (Figure 4C), the difference among the samples became more pronounced. After 5 h of heating, AP-A retained 64.42% of anthocyanins, compared with 48.52% in the CK group, followed by CP-A (58.54%) and BP-A (55.81%). These results indicate that complexation with pectin helped improve the thermal retention of bilberry anthocyanins under the tested conditions, which is consistent with previous reports on anthocyanin-polysaccharide systems [40]. Similarly, Fu et al. (2023) [19] further demonstrated that pectin complexation increased the thermal stability of isolated anthocyanins, with the pectin–anthocyanin system exhibiting greater resistance to heat-induced pigment deterioration than free anthocyanins.

The accelerated degradation observed at higher temperatures is generally associated with anthocyanin structural transformation and degradation reactions, including glycosidic bond cleavage, hydration of the flavylium cation, and formation of colorless or brown degradation products [46]. In the present study, the higher retention observed in the pectin-complexed samples may be related to the interaction between anthocyanins and pectin chains, as suggested by the FT-IR results. Hydrogen bonding, electrostatic interactions, and hydrophobic interactions may contribute to the association between anthocyanins and pectin, thereby reducing the exposure of anthocyanins to the surrounding aqueous environment during heating [40,46]. Among the three complexes, AP-A showed the highest retention, followed by CP-A and BP-A. This order was consistent with the binding percentages shown in Figure 1 and the compositional differences discussed in Section 3.1.2. Therefore, these results suggest that the pectin source and compositional features may influence the thermal stability of pectin–anthocyanin complexes, although further quantitative binding analysis would be needed to clarify the detailed mechanism.

#### 3.4.2. Photostability and Degradation Kinetics

**Figure 5:**
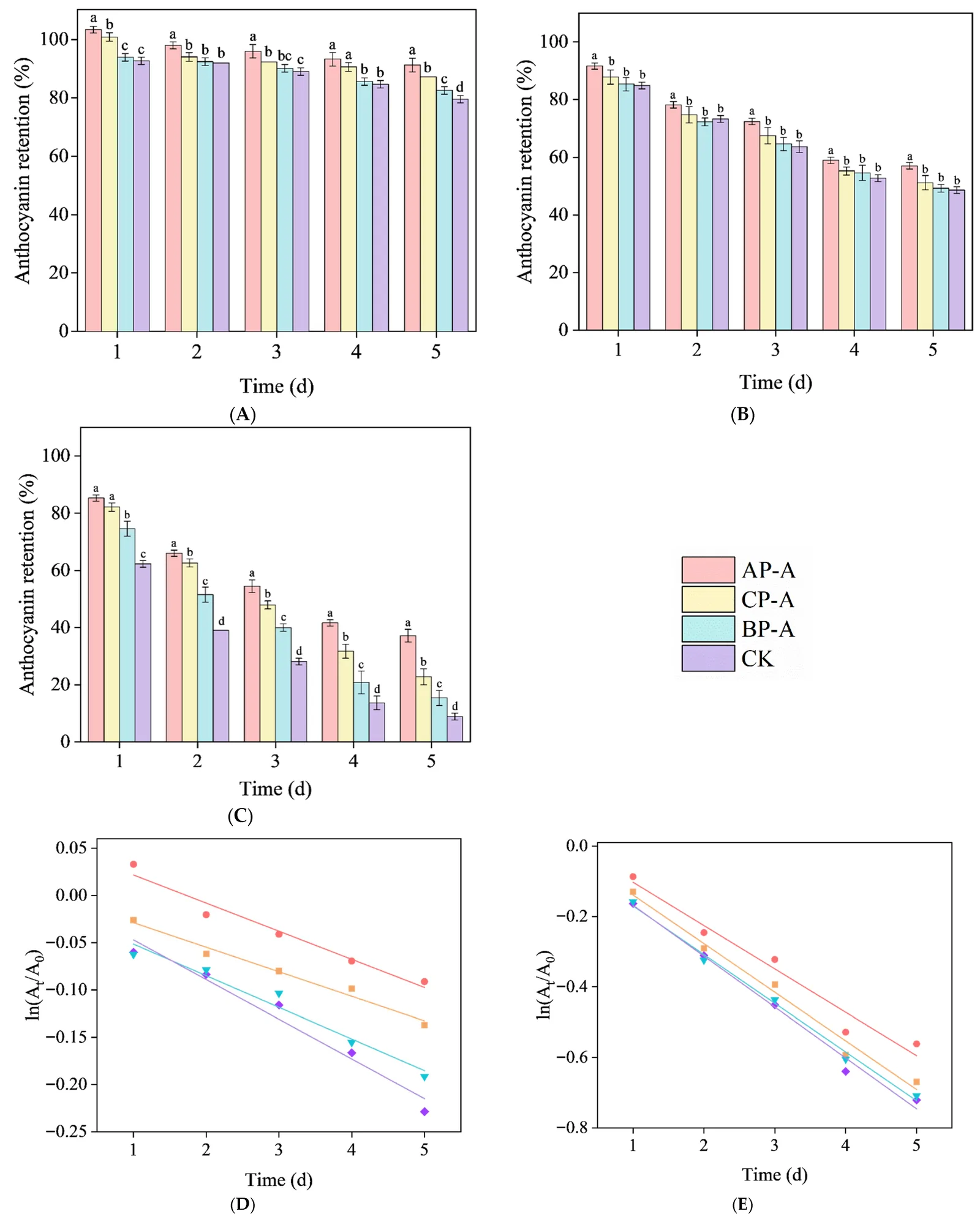

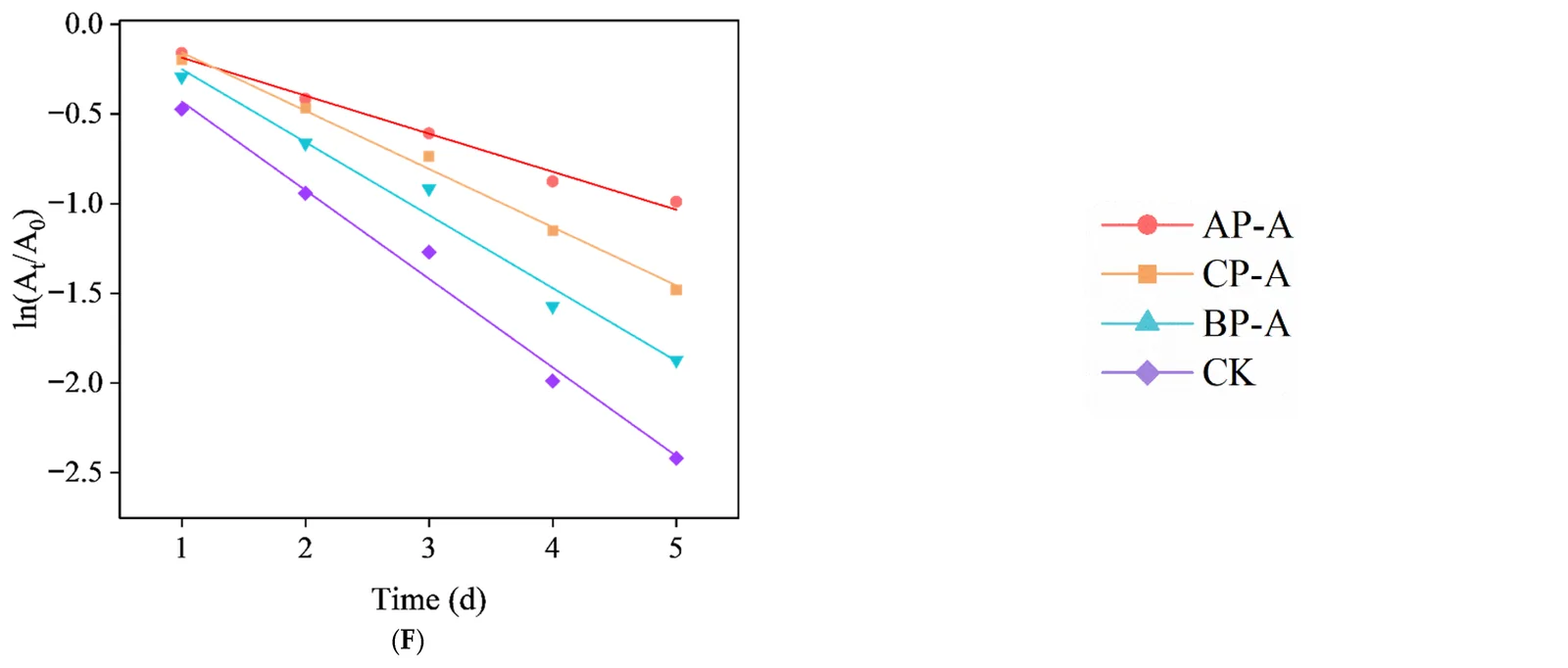
Effect of different light conditions on anthocyanin retention and degradation kinetics of free bilberry anthocyanins and pectin–anthocyanin complexes. (**A**,**D**) Light-protected storage; (**B**,**E**) incandescent light; and (**C**,**F**) indoor natural light. (**A**–**C**) Anthocyanin retention; (**D**–**F**) corresponding first-order kinetic plots. Different lowercase letters indicate significant differences among samples at the same treatment time (*p* < 0.05).

The photostability of free and complexed bilberry anthocyanins was monitored under three storage conditions: light-protected storage, incandescent light exposure, and indoor natural light exposure (Figure 5). During the 5-day observation period, anthocyanin retention decreased in all samples, and the extent of degradation varied with the light condition. The light-protected samples showed higher retention, whereas indoor natural light resulted in a greater decrease in anthocyanin retention. After 5 days of exposure to indoor natural light, the retention in the CK group decreased to 8.90%, whereas the pectin complexes retained higher levels: 37.18% for AP-A, 22.76% for CP-A, and 15.38% for BP-A (Figure 5C). Under indoor natural light, the calculated half-life (t_1/2_) increased from 1.48 d in the CK group to 3.51, 2.75, and 2.04 d in the AP-A, CP-A, and BP-A systems, respectively (Table S2). These results suggest that pectin complexation improved the photostability of bilberry anthocyanins under the tested light conditions, with the retention following the order AP-A > CP-A > BP-A > CK.

The degradation process under the tested light conditions was fitted using a first-order kinetic model, as shown by the relationship between ln(A_t_/A_0_) and time (Figure 5D–F). The degradation rate constants (*k*) differed among the samples, and AP-A showed a lower k value than the other groups under natural light (Table S2), indicating a slower decrease in anthocyanin retention. Light exposure has been reported to accelerate anthocyanin degradation through structural transformation and pigment decomposition reactions [47]. Therefore, the slower degradation observed in the pectin-complexed systems may be associated with the stabilizing effect of pectin–anthocyanin interactions. The improved photostability of the pectin complexes may be partly attributed to non-covalent interactions between anthocyanins and pectin. These interactions may help maintain anthocyanins in a more stable local environment and reduce their susceptibility to light-induced degradation [19,37]. Jiang et al. [48] reported that pectin with a high degree of esterification reduced the light degradation rate of cyanidin-3-O-glucoside from 0.05 to approximately 0.03 h^−1^, thereby improving its light stability. Although direct quantitative comparison is limited by differences in illumination conditions and anthocyanin systems, a similar protective trend was observed in the present study. These findings indicate that the selection of pectin source may be relevant for improving anthocyanin stability in light-sensitive food systems.

#### 3.4.3. Metal Ions

**Figure 6:**
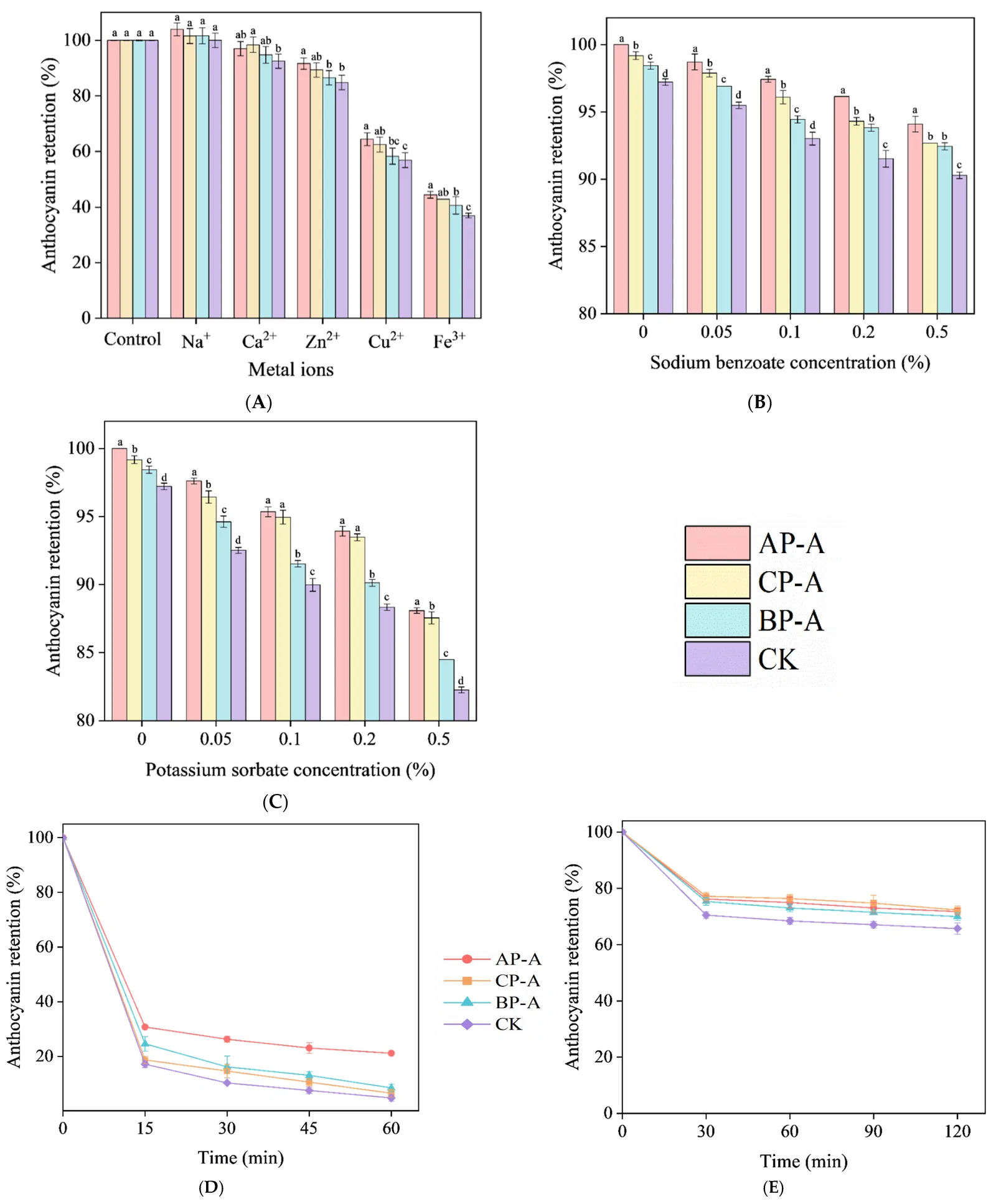
Effects of different chemical treatments on the anthocyanin retention of free bilberry anthocyanins and pectin–anthocyanin complexes. (**A**) Metal ion treatment; (**B**) sodium benzoate treatment; (**C**) potassium sorbate treatment; (**D**) H_2_O_2_ treatment; and (**E**) Na_2_SO_3_ treatment. Different lowercase letters indicate significant differences among samples at the same concentration (*p* < 0.05).

The effects of different metal ions on the stability of bilberry anthocyanins and their complexes are shown in Figure 6A. The retention varied depending on the ion species. Na^+^ showed a limited effect on anthocyanin retention, whereas Ca^2+^ and Zn^2+^ caused moderate decreases. In contrast, Cu^2+^ and Fe^3+^ were associated with lower retention levels. In the presence of Fe^3+^, retention percentages for AP-A, CP-A, BP-A, and the CK were 40.28%, 39.29%, 38.54%, and 37.00%, respectively. Although the pectin complexes showed slightly higher retention than CK, the protective effect was relatively limited under Fe^3+^ treatment. These observations are consistent with previous reports that transition metal ions, particularly Cu^2+^ and Fe^3+^, can accelerate anthocyanin degradation [49].

The destabilizing effect of transition metal ions may be related to metal-anthocyanin complexation and metal-catalyzed oxidation reactions, which can promote pigment degradation. The effect of Zn^2+^ observed in this study differed from some previous reports [50], suggesting that the influence of metal ions may depend on anthocyanin composition, pH, and the surrounding matrix. The slightly higher retention of the pectin complexes, particularly AP-A, may be associated with the metal-binding ability of pectic carboxyl groups, which could reduce the direct interaction between metal ions and anthocyanins [51]. Previous studies have likewise shown that pectin association can improve the stability of cyanidin-3-O-glucoside under metal-ion stress, including enhanced stability in the presence of Cu^2+^ after interaction with RG-I pectin [52]. These results suggest that transition metal ions should be considered during the processing and storage of anthocyanin-containing products, especially when iron or copper exposure is possible.

#### 3.4.4. Food Preservatives

The effects of sodium benzoate and potassium sorbate on bilberry anthocyanins were evaluated at different concentrations (Figure 6B,C). Across all groups, anthocyanin retention decreased with increasing preservative concentration. At 0.5% sodium benzoate, the retention percentages of AP-A, CP-A, BP-A, and CK were 94.87%, 92.68%, 92.29%, and 90.42%, respectively. At the same concentration of potassium sorbate, the retention values were lower, reaching 88.10%, 87.41%, 84.50%, and 82.38%, respectively. These results indicate that both preservatives affected anthocyanin stability, with potassium sorbate showing a stronger destabilizing effect than sodium benzoate under the present conditions. This trend is consistent with the findings of Moldovan and David [53], who reported that the stability of anthocyanins in aqueous systems can be affected by sodium benzoate and potassium sorbate. A similar result was reported for blackcurrant anthocyanins, where potassium sorbate was associated with accelerated anthocyanin degradation during storage, further indicating that preservative type can influence anthocyanin stability [54].

The decrease in anthocyanin retention in the presence of preservatives may be related to changes in the chemical environment of the pigment solution and interactions between preservative species and anthocyanins. Compared with CK, the pectin complexes maintained higher retention across the tested concentrations, following the order AP-A > CP-A > BP-A > CK. This result suggests that pectin complexation may partly reduce preservative-associated anthocyanin loss. The higher retention observed in AP-A may be related to its higher binding percentage and the compositional characteristics of AP. Taken together, these results indicate that the influence of food preservatives on anthocyanin stability may depend on both preservative type and the surrounding pectin matrix.

#### 3.4.5. Oxidative and Reductive Agents

The chemical stability of free and complexed bilberry anthocyanins was further evaluated using H_2_O_2_ and Na_2_SO_3_ to represent oxidative and reductive conditions (Figure 6D,E). Treatment with 0.5% H_2_O_2_ caused a rapid decrease in anthocyanin retention. After 60 min, the retention in the CK group decreased to 4.79%, whereas AP-A retained 27.68%, followed by BP-A (8.46%) and CP-A (6.50%). These results show that AP-A retained a higher proportion of anthocyanins than the other samples under H_2_O_2_ treatment. H_2_O_2_-induced anthocyanin degradation has been associated with oxidation reactions involving the flavylium structure, which can lead to loss of color and pigment degradation [55,56]. The higher retention of AP-A may be related to stronger pectin–anthocyanin association suggested by its higher binding percentage, which could reduce the accessibility of anthocyanins to oxidative attack to some extent. Comparable findings have been reported for anthocyanin-containing polysaccharide systems, in which polysaccharide association contributed to greater anthocyanin retention under oxidative conditions [57].

Treatment with 0.5% Na_2_SO_3_ also caused a rapid decline in anthocyanin retention, which is consistent with the known bleaching effect of sulfites on anthocyanins. Bisulfite ions can react with flavylium cations to form colorless sulfonate adducts [55,58]. After 120 min, the pectin-complexed systems showed higher retention than CK, with the order AP-A > CP-A > BP-A > CK. This suggests that pectin complexation may provide partial protection against sulfite-induced discoloration, although the protective effect was limited under the tested Na_2_SO_3_ condition. The greater anthocyanin retention observed in the pectin-complexed systems under these chemical stresses is in agreement with previous studies showing that polysaccharide association can enhance anthocyanin stability under adverse chemical conditions [19]. Overall, these results indicate that pectin complexation, particularly with AP, may improve the retention of bilberry anthocyanins under selected oxidative and reductive stresses, but the extent of protection depends on the type of chemical stress.

### 3.5. Comparative Analysis of Antioxidant Activities

**Figure 7:**
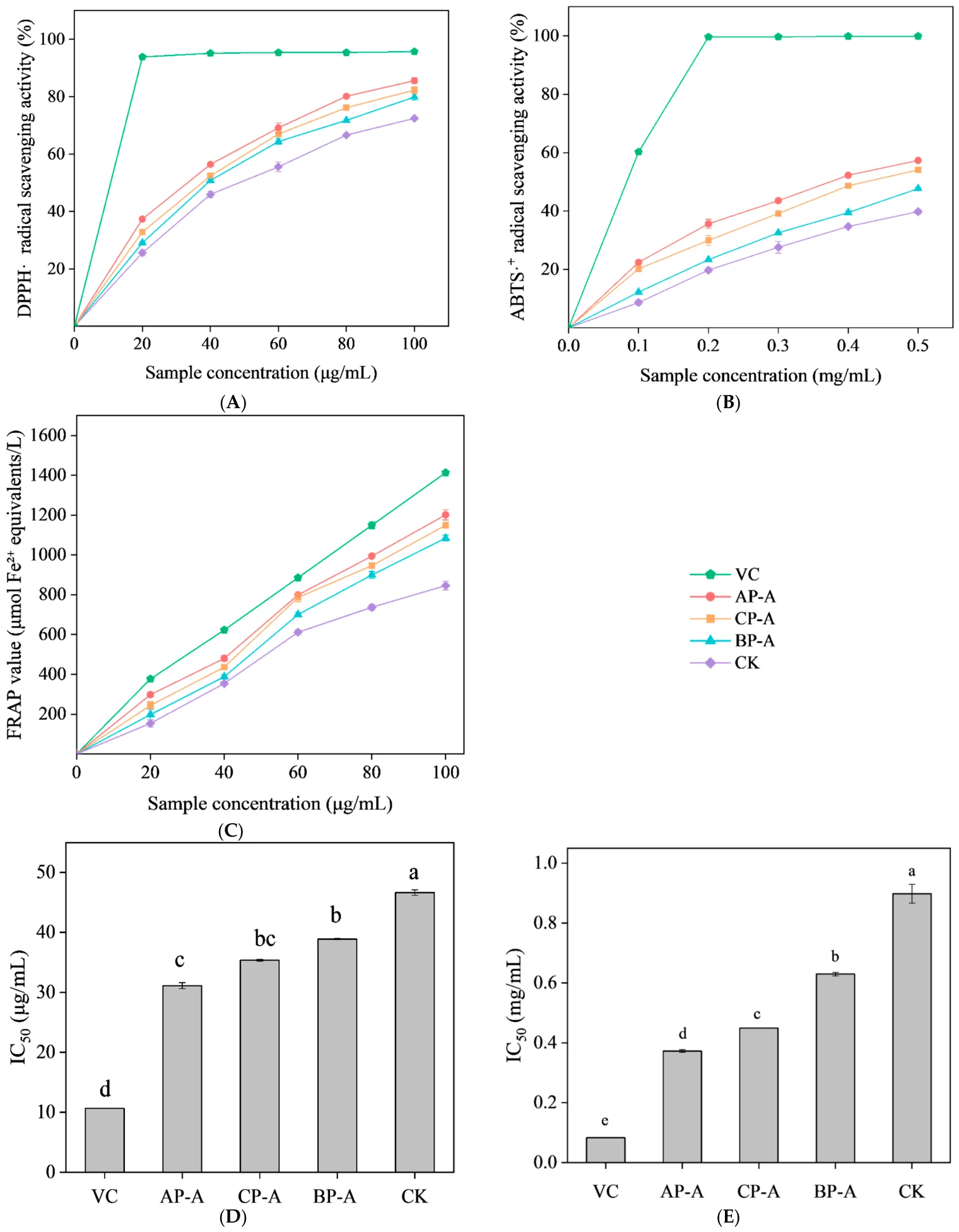
Antioxidant activities of free bilberry anthocyanins and their pectin complexes. (**A**) DPPH• radical scavenging activity; (**B**) ABTS•^+^ radical scavenging activity; (**C**) ferric reducing antioxidant power (FRAP); (**D**) IC_50_ values for DPPH• radical scavenging activity; and (**E**) IC_50_ values for ABTS•^+^ radical scavenging activity. VC, ascorbic acid; CK, free bilberry anthocyanins. Different lowercase letters indicate significant differences among samples at the same concentration (*p* < 0.05).

To obtain a more comprehensive evaluation of antioxidant performance, DPPH, ABTS, and FRAP assays were employed because they provide complementary information. DPPH and ABTS reflect radical-scavenging capacity under different reaction conditions, whereas FRAP evaluates reducing power. Their combined use therefore enables a broader assessment of the antioxidant behavior of the pectin–anthocyanin systems from a food application perspective. The antioxidant performance of the samples is shown in Figure 7. Across all tested concentrations, a concentration-dependent response was observed, with the radical scavenging activities and reducing power increasing with increasing sample concentrations. Under the current assay conditions, the pectin–anthocyanin complex systems showed higher antioxidant activities than the free anthocyanin sample at the corresponding sample mass concentrations, following the order VC > AP-A > CP-A > BP-A > CK. Specifically, the IC_50_ values for DPPH radical scavenging showed that free anthocyanins exhibited a higher IC_50_ value (46.640 ± 0.44 μg/mL), whereas the AP-A complex showed a lower IC_50_ value (31.12 ± 0.49 μg/mL) (Figure 7D). A similar trend was observed in the ABTS assay (Figure 7E), where the IC_50_ of free anthocyanins (0.90 ± 0.03 mg/mL) was reduced to 0.372 ± 0.004 mg/mL in the AP-A system. These findings are consistent with previous reports suggesting that pectin-containing matrices can help maintain or improve the antioxidant performance of anthocyanin-rich extracts by reducing anthocyanin degradation or improving their stability in the assay system [19,59]. Furthermore, the reducing capacity measured by the FRAP assay (Figure 7C) showed that the pectin–anthocyanin complexes had higher reducing capacity than free anthocyanins, with AP-A exhibiting the highest FRAP values among the three complexes. This result may be related to the higher anthocyanin retention and complex stability observed for AP-A in the preceding sections [60].

A possible explanation for the improved antioxidant performance is that the overall pectin–anthocyanin systems may provide a more favorable local environment for anthocyanins during the assays, while non-covalent association between anthocyanins and pectin may contribute to this effect. This effect may be associated with the compositional characteristics of the pectin matrices discussed in preceding sections, which could contribute to a more favorable association between anthocyanins and the pectin matrix, thereby partly limiting anthocyanin exposure to oxidative conditions [19,38]. Furthermore, the changes in crystalline characteristics of the pectin–anthocyanin complexes, as suggested by the XRD results in Section 3.3.2, may also be related to the different antioxidant responses observed among the complexes. The differential efficacy observed among AP-A, CP-A, and BP-A was consistent with the trends observed in binding percentage and stability assays, suggesting that the monosaccharide-derived compositional descriptors of pectin, particularly the neutral sugar side-chain indicator, may be associated with the antioxidant activity of the complexes [22,38]. According to Fu et al. (2023) and Dong et al. [19,61], anthocyanin-polysaccharide association can help maintain antioxidant capacity under environmental stress. Overall, these results suggest that apple pectin may represent a suitable matrix for improving the stability and antioxidant activity of bilberry anthocyanins in food systems.

## 4. Conclusions

This study compared apple pectin, citrus pectin, and blueberry pectin as food-grade matrices for stabilizing bilberry anthocyanins. Under the selected complexation conditions, the binding percentage and anthocyanin retention performance under the tested stability assays generally followed the order AP-A > CP-A > BP-A, suggesting that differences in the compositional characteristics of the tested commercial pectin samples may be associated with anthocyanin binding and stabilization behavior. FT-IR, XRD, and SEM analyses indicated changes in functional group regions, diffraction characteristics, and surface morphology after complex formation, which were consistent with the involvement of non-covalent interactions. Among the three complexes, AP-A showed relatively higher retention of bilberry anthocyanins under thermal, light, metal ion, preservative, oxidative, and reductive stresses, and also maintained higher antioxidant activity than free anthocyanins. These findings provide comparative evidence for selecting suitable pectin sources to improve the stability of anthocyanin-rich food ingredients. However, the present study mainly compared pectins based on their source and monosaccharide composition. Future studies are warranted to provide more comprehensive structural characterization of pectins, quantitative analysis of pectin–anthocyanin interactions, and validation in real food matrices.

## Figures and Tables

**Table 1:** Monosaccharide composition of three commercial pectin samples.

	Monosaccharide Content (μg/mg)							Derived Compositional Descriptors	
	Fuc	Ara	Rha	Gal	Glc	Xyl	GalA	Rha/GalA	(Gal + Ara)/Rha
Apple pectin	0.40 ± 0.01	2.92 ± 0.05	10.74 ± 0.13	19.21 ± 0.15	146.87 ± 1.94	8.06 ± 0.12	23.86 ± 0.34	0.45	2.06
Citrus pectin	0.63 ± 0.003	1.78 ± 0.01	1.96 ± 0.02	3.47 ± 0.04	37.23 ± 0.65	2.00 ± 0.03	18.47 ± 0.27	0.11	2.68
Blueberry pectin	0.83 ± 0.01	19.61 ± 0.09	10.67 ± 0.21	21.77 ± 0.14	109.33 ± 2.14	0.75 ± 0.01	28.76 ± 0.35	0.37	3.88

Notes: Fuc, fucose; Ara, arabinose; Rha, rhamnose; Gal, galactose; Glc, glucose; Xyl, xylose; GalA, galacturonic acid. Monosaccharide contents are expressed as mean ± standard deviation (SD) of three replicate samples for each pectin type (*n* = 3). The Rha/GalA and (Gal + Ara)/Rha ratios were calculated from the mean monosaccharide contents and used as comparative compositional descriptors.

## Data Availability

The original contributions presented in this study are included in the article/Supplementary Materials. Further inquiries can be directed to the corresponding author.

## Supplementary Materials

The following supporting information can be downloaded at https://www.mdpi.com/article/10.3390/foods15183312/s1: Figure S1: UV-Vis absorption spectrum of bilberry anthocyanin extract. Table S1: Tentative identification of representative anthocyanins in bilberry extract by HPLC-MS/MS. Table S2: Degradation kinetic parameters of free bilberry anthocyanins and pectin–anthocyanin complexes under different light conditions.
