# Supplementary material for: Comparative Effects of Fruit-Derived Pectins on the Stability and Antioxidant Activity of Bilberry Anthocyanin–Pectin Complexes
Source: Foods. 2026 Sep 18;15(18):3312. doi: 10.3390/foods15183312 (PMC13606454; doi:10.3390/foods15183312)
Supplement: Supplementary file 1 [file foods-15-03312-s001.zip › foods-4510631-supplementary.pdf]

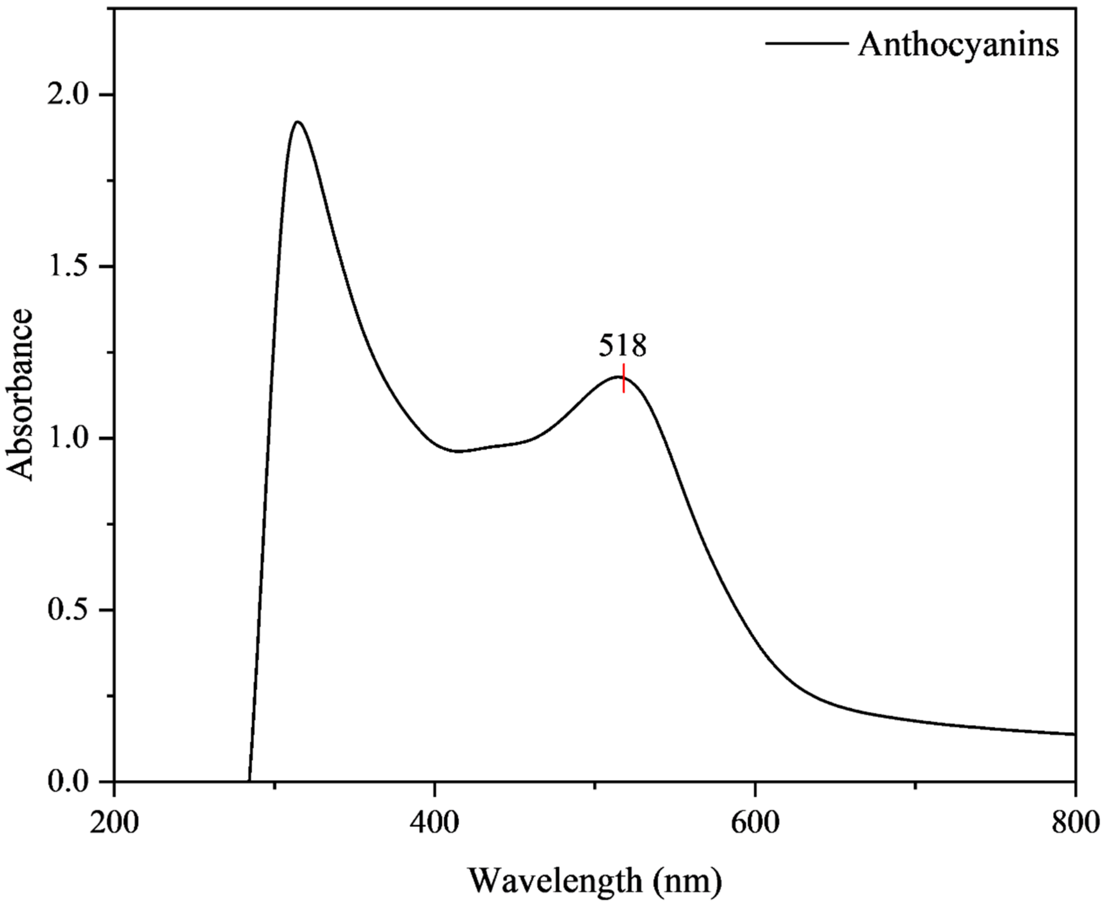

Figure S1. UV-Vis absorption spectrum of bilberry anthocyanin extract.

Table S1 Tentative identification of representative anthocyanins in bilberry extract by HPLC-MS/MS.

Peak No.	Retention time (min)	Molecular formula	Molecular ion (m/z)	Fragment ion (m/z)	Tentatively assigned compound
1	5.99	$C_{21}H_{21}O_{11}$	449.11	287.05	Cyanidin-3-O-glucoside
2	6.62	$C_{27}H_{31}O_{15}$	595.16	287.05	Cyanidin-3-O-rutinoside
3	7.35	$C_{22}H_{23}O_{11}$	463.12	301.07	Peonidin-3-O-glucoside

Table S2 Degradation kinetic parameters of free bilberry anthocyanins and pectin-anthocyanin complexes under different light conditions.

Light condition	Sample	Equation	Degradation rate constant k (d^{-1})	R^2	Half-life $t_{1/2}$ (d)
Indoor natural light	CK	y=-0.4694x+0.0638	0.4694	0.9879	1.48
	BP-A	y=-0.3394x+0.1570	0.3394	0.9799	2.04
	CP-A	y=-0.2519x+0.1681	0.2519	0.9926	2.75
	AP-A	y=-0.1972x+0.0267	0.1972	0.9872	3.51
Incandescent light	CK	y=-0.1546x-0.0239	0.1546	0.9898	4.48
	BP-A	y=-0.1517x-0.0319	0.1517	0.9942	4.57
	CP-A	y=-0.1377x-0.0010	0.1377	0.9856	5.03
	AP-A	y=-0.1123x+0.0203	0.1123	0.9641	6.17
Light-protected condition	CK	y=-0.0456x-0.0051	0.0456	0.9640	15.20
	BP-A	y=-0.0427x-0.0180	0.0427	0.9633	16.23
	CP-A	y=-0.0271x-0.0029	0.0271	0.9796	25.58
	AP-A	y=-0.0197x+0.0515	0.0197	0.9642	35.19
